# Investigation of *Cryptosporidium* infection in a broad range of hosts in northern China

**DOI:** 10.1186/s13071-025-07152-9

**Published:** 2025-11-26

**Authors:** Ziheng Liu, Jing Yang, Jiushikun Juman, Nannan Cui, Ligu Mi, Sándor Hornok, Guoyu Zhao, Quan Liu, Yuanzhi Wang

**Affiliations:** 1 Key Laboratory for Prevention and Control of Emerging Infectious Diseases and Public Health Security of the XPCC, School of Medicine, Shihezi University, Shihezi, 832003 Xinjiang Uygur Autonomous Region China; 2 NHC Key Laboratory of Prevention and Treatment of Central Asia High Incidence Diseases, Shihezi University, Shihezi, 832003 Xinjiang Uygur Autonomous Region China; 3 Key Laboratory of Vector-Borne Infectious Diseases, Center for Disease Control and Prevention, Urmqi, Xinjiang Uygur Autonomous Region China; 4 Department of Parasitology and Zoology, University of Veterinary Medicine, Budapest, Hungary; 5 HUN-REN-UVMB Climate Change: New Blood-Sucking Parasites and Vector-Borne Pathogens Research Group, Budapest, Hungary

**Keywords:** *Cryptosporidium*, Wildlife, PCR, Northern China

## Abstract

**Background:**

*Cryptosporidium* infection occurs in humans, domestic animals, and wildlife. To date, at least 49 species and 120 genotypes have been identified. Hitherto, molecular identification of *Cryptosporidium* species in wildlife has seldom been reported in China.

**Methods:**

During 2014–2025, a total of 1855 small intestinal or fecal specimens were collected from 1500 mammals, 121 reptiles, and 234 birds in Xinjiang Uygur Autonomous Region (XUAR) and Inner Mongolia Autonomous Region (IMAR), northern China. The identification of each animal species was based on morphological characteristics and mitochondrial gene amplification. Detection of *Cryptosporidium* species was performed by amplifying part of the small subunit (*SSU*) ribosomal RNA (*rRNA*) gene. The 60 kDa glycoprotein (*GP60*) gene was used to confirm their species and subtypes.

**Results:**

The samples were collected from 39 mammalian, 6 reptilian, and 30 avian species. In these samples, the average rate of infection with *Cryptosporidium* species was 8.09% (150/1855). In total, 18 known *Cryptosporidium* species and genotypes were identified, including *Cryptosporidium hominis*, *Cryptosporidium ubiquitum*, *Cryptosporidium muris*, *Cryptosporidium canis*, *Cryptosporidium felis*, *Cryptosporidium equi*, *Cryptosporidium proventriculi*, *Cryptosporidium ryanae*, *Cryptosporidium rubeyi*, chipmunk genotype V, vole genotype III, vole genotype V, muskrat genotype I, bat genotype IV, yak genotype, deer genotype, goose genotype I, and one unnamed *Cryptosporidium* sp. In addition, a novel genotype, here designated as *Cryptosporidium* Mongolian pika genotype, was identified in the Mongolian pika (*Ochotona pallasi*).

**Conclusions:**

Investigation of *Cryptosporidium* infection was carried out by screening 75 animal species. Overall, 19 *Cryptosporidium* species and genotypes were detected, including a novel genotype in Mongolian pika and first-time diagnosis of this infection in several rodent species (e.g., red-cheeked ground squirrels, great gerbils, northern mole voles, and Libyan jirds).

**Graphical Abstract:**

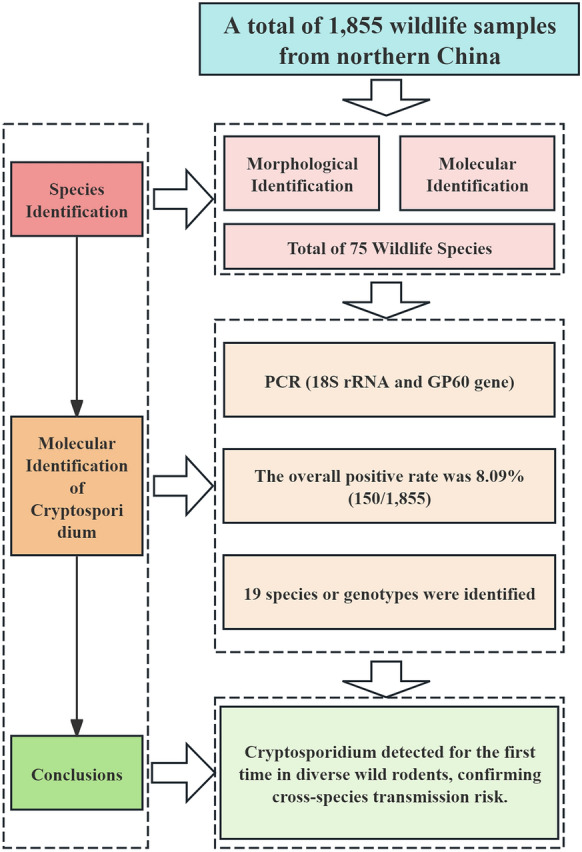

**Supplementary Information:**

The online version contains supplementary material available at 10.1186/s13071-025-07152-9.

## Background

*Cryptosporidium* species (Apicomplexa: Sporozoasida: Gregarinasina) infect a broad range of vertebrates, including domestic animals, wildlife, and humans, implying zoonotic transmission [1]. To date, at least 49 species and 120 genotypes have been reported globally [2–4]. Infection manifests as watery diarrhea, abdominal pain, nausea, vomiting, and fever, with more severe outcomes in immunocompromised individuals due to conditions such as human immunodeficiency virus/acquired immune deficiency syndrome (HIV/AIDS), cancer, immunosuppressive therapies, or malnutrition [5, 6].

Wildlife plays multifaceted roles in the transmission of *Cryptosporidium*, serving both as natural reservoirs and key intermediaries in transmission chains [7]. Multiple *Cryptosporidium* species and genotypes were not only detected in wildlife but are also considered to be emerging zoonotic pathogens, as exemplified by *Cryptosporidium ubiquitum* and *Cryptosporidium hominis* [8, 9]. In China, human cryptosporidiosis was documented in 27 provinces or autonomous regions [10]. At the same time, domestic livestock, pets, and zoo animals were also reported to harbor these protozoan parasites [11–13]. However, large-scale investigation of *Cryptosporidium* in a broad spectrum of wildlife species is still lacking in northern China.

Xinjiang Uygur Autonomous Region (XUAR) and Inner Mongolia Autonomous Region (IMAR), covering 1.6749 and 1.1830 million km^2^, respectively, are listed as the first and third largest provincial-level administrative divisions in China. These regions, located in northern China, harbor diverse natural landscapes (e.g., forests, meadows, deserts, glacial zones, and lacustrine areas) and extensive territories, which provide suitable habitats for abundant wildlife populations, including endemic species [14]. To characterize *Cryptosporidium* infections in wildlife and to assess preliminarily cross-species transmission risks in northern China, a large-scale molecular investigation of *Cryptosporidium* species was performed involving 1500 wild mammals, 121 reptiles, and 234 birds in these regions.

## Methods

### Sample collection and identification

During the period from May 2015 to May 2025, 1709 small intestinal samples were obtained from 273 pastured donkeys (imported from Kyrgyzstan) and 1436 wildlife animals in XUAR and IMAR, as well as 146 fecal samples (from 10 zoo mammals and 136 migratory birds). All animals involved in the study were euthanized under the supervision of veterinarians, including anesthesia and intravenous injection of pentobarbital. Identification of host species was based on key morphological characteristics [15–17] and amplification of mitochondrial genetic markers, including the 910-base pair (bp)-long 16S ribosomal RNA (*16S rRNA*) fragment for bird tissues [18], the 924-bp-long displacement loop (D-loop) mitochondrial DNA (mtDNA) for bird feces [19], the 650-bp-long cytochrome *c* oxidase subunit I (*COX1*) for reptiles [20], and a 1242-bp-long cytochrome B (*CytB*) sequence for rodents (Additional File 2) [21]. The corresponding sequences were deposited in the GenBank database (*16S rRNA*: PQ394778–PQ394785, PQ451723–PQ451727, and PQ459365; *COX*1: PQ451734–PQ451736; D-loop mtDNA: PQ373942–PQ373943, PQ393132–PQ393138, and PQ474771; *CytB*: PQ450150–PQ450159, PQ450161–PQ450162, PQ450165, PQ450168, PQ474772–PQ474773, and PQ581939).

### DNA extraction

Genomic DNA was extracted from each small intestinal sample using the TIANamp Genomic DNA Kit (TIANGEN, Beijing, China) following the manufacturer’s instructions. The genomic DNA from each fecal sample was extracted using the EasyPure Stool Genomic DNA Kit (TRANS, Beijing, China). DNA quantity was assessed on a NanoDrop 2000 spectrophotometer (Termo Fisher Scientifc, Waltham, MA, USA). Samples with a DNA concentration of at least 30 ng/μl could be used to detect *Cryptosporidium*.

### Genotyping and subtyping of *Cryptosporidium* spp.

Part of the *SSU rRNA* gene (length: 826–864 bp) was amplified by a nested polymerase chain reaction (nPCR) to detect *Cryptosporidium* spp. in each extracted DNA sample [22]. Reagent-grade water was used as negative control. All nPCR products were sequenced by Youkang Biotechnology Co., Ltd. (Urumqi, China). In addition, the 60 kDa glycoprotein (*GP60*) gene was used to confirm *Cryptosporidium* species and subtypes [23]. The primers and PCR conditions are shown in Additional File 2.

### Sequencing and data analyses

Sequences obtained in this study were compared with GenBank data by the nucleotide BLAST method (http://www.ncbi.nlm.nih.gov/blast/) and then aligned and analyzed with reference sequences downloaded from GenBank. A total of 44 *Cryptosporidium* sequences were deposited in the GenBank database (*SSU rRNA*: PQ568971.2, PQ569063–PQ569074, PQ569075.2–PQ569083, PQ865466, PQ865468–PQ865469, PV794553.2–PV794558, PV794559.2–PV794562, PX116791–PX116794, and PX248577; *GP60*: PQ588454, PQ606086–PQ606087, and PV800351). Phylogenetic trees were constructed on the basis of the T92 + G + I (Tamura 3-parameter model, gamma distributed with invariant sites) model using the maximum-likelihood method in MEGA 7.0 (http://www.megasoftware.net/) software (Fig. 2).

## Results

### Host identification

**Fig. 1:**
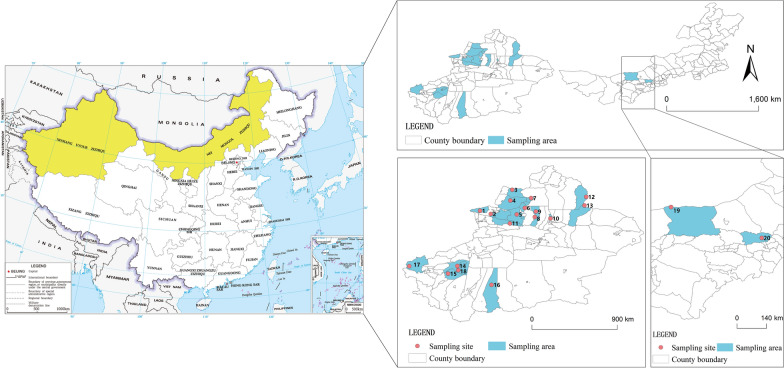
The small intestine samples of wild animals and the location where feces were collected in this study

A total of 1855 small intestinal or fecal samples were included in this study, obtained from 39 mammalian, 30 avian, and 6 reptilian species (Additional File 1). The place of their geographical origin and other data are shown in Fig. 1.

### Prevalence of *Cryptosporidium* spp.

The overall prevalence of *Cryptosporidium* species in these samples was 8.1% (150/1,855), with a prevalence of 9.5% (142/1500) in mammals and 3.4% (8/234) in birds. Infection with *Cryptosporidium* was not detected in reptiles.

Among the small intestinal samples from 1500 mammals, the highest infection rates were observed in the orders Rodentia (12.3%, 98/794) and Carnivora (7.7%, 3/39). Among rodents, there was a significant difference in the prevalence of *Cryptosporidium* infection between Sciuridae (14.6%, 62/424) and Muridae (7.9%, 24/303) (*X*^2^ = 9.33, *df* = 1; *P* < 0.01).

### Species and genotypes of *Cryptosporidium* spp.

**Fig. 2:**
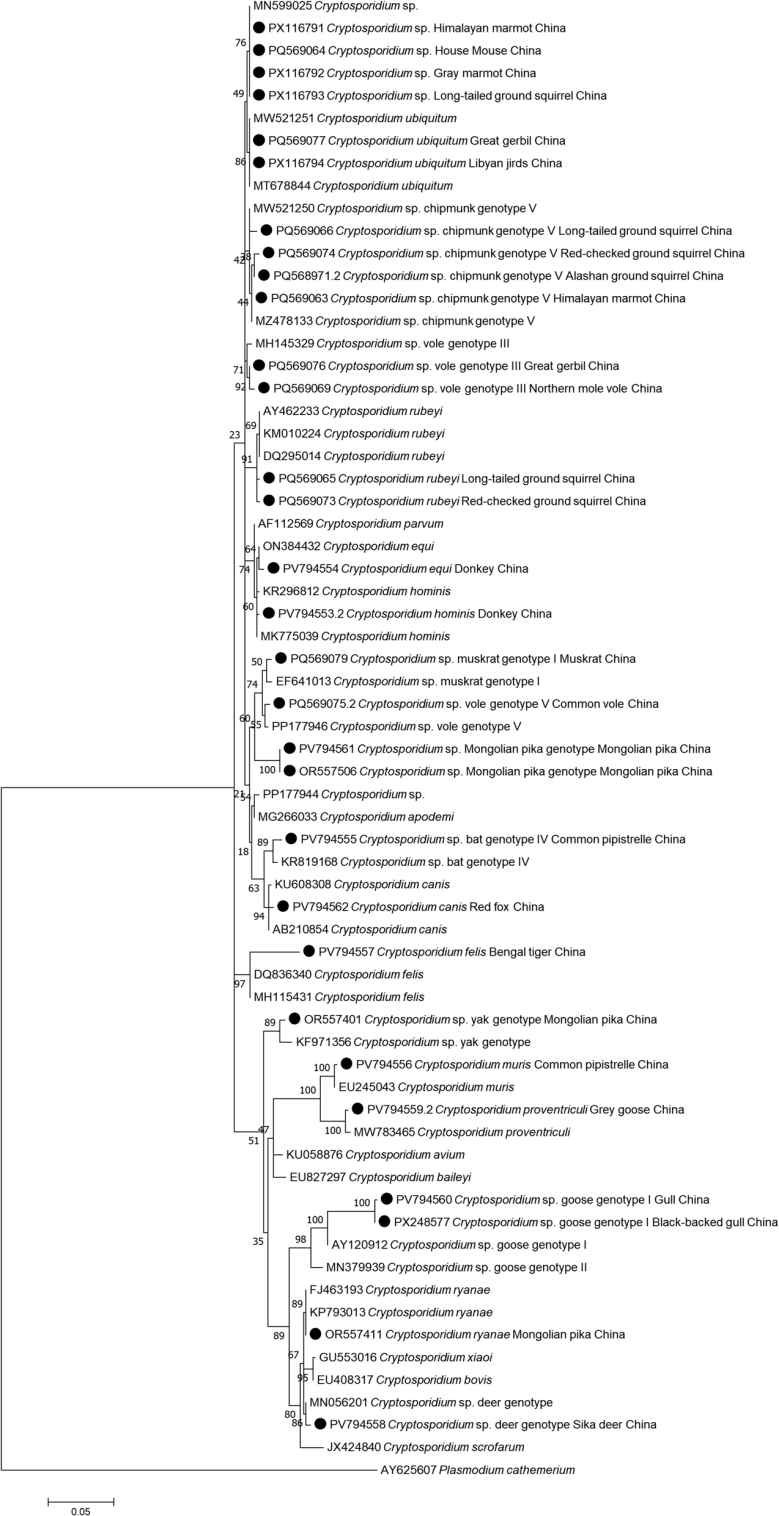
Phylogenetic tree of *Cryptosporidium* species and genotypes was conducted using the maximum-likelihood method based on *SSU rRNA* gene sequences under the T92 + G + I (Tamura 3-parameter model, gamma distributed with invariant sites) model with 1000 bootstrap replicates. Novel sequences identified in this study are denoted by black circles and labeled with GenBank accession numbers, host species, and country of origin. *Plasmodium cathemerium* was used as an outgroup

Molecular analysis indicated 18 distinct species/genotypes, including *C*. *hominis* (*n* = 6), *C*. *ubiquitum* (*n* = 20), *Cryptosporidium muris* (*n* = 3), *Cryptosporidium canis* (*n* = 2), *Cryptosporidium felis* (*n* = 1), *Cryptosporidium ryanae* (*n* = 2), *Cryptosporidium rubeyi* (*n* = 25), *Cryptosporidium equi* (*n* = 7), *Cryptosporidium proventriculi* (*n* = 6), chipmunk genotype V (*n* = 22), vole genotype III (*n* = 4), vole genotype V (*n* = 7), muskrat genotype I (*n* = 3), bat genotype IV (*n* = 12), yak genotype (*n* = 6), deer genotype (*n* = 1), goose genotype I (*n* = 2), and *Cryptosporidium* sp. (*n* = 17) (presented in Table 1 and Additional File 1). The above species and genotypes of *Cryptosporidium* were confirmed by phylogenetic analysis of the *SSU rRNA* gene. The phylogenetic tree that was constructed using the *SSU rRNA* gene is shown in Fig. 2.

**Table 1:** *Cryptosporidium* species/genotypes detected in this study

Host species	Gene	*Cryptosporidium* species/genotype detected	GenBank no.	Similar sequences, percentage identity, and GenBank no.	Source reference of the similar sequence
*Marmota himalayana*	*SSU rRNA*	*Cryptosporidium* sp.	PX116791	*Cryptosporidium* sp. 798/800 (99.75%) MN599025	García-Livia K, Martín-Alonso A, Foronda P. Diversity of *Cryptosporidium* spp. in wild rodents from the Canary Islands, Spain. *Parasit Vectors*. 2020;13:445
*Marmota himalayana*	*SSU rRNA*	*Cryptosporidium* sp. Chipmunk genotype V	PQ569063	*Cryptosporidium* sp. Chipmunk genotype V 793/796 (99.62%) MZ478133	Xu J, Liu H, Jiang Y, Jing H, Cao J, Yin J, et al. Genotyping and subtyping of *Cryptosporidium* spp. and *Giardia duodenalis* isolates from two wild rodent species in Gansu Province, China. *Sci Rep*. 2022;12:12,178
*Marmota baibacina*	*SSU rRNA*	*Cryptosporidium* sp.	PX116792	*Cryptosporidium* sp. 798/799 (99.87%) MN599025	García-Livia K, Martín-Alonso A, Foronda P. Diversity of *Cryptosporidium* spp. in wild rodents from the Canary Islands, Spain. *Parasit Vectors*. 2020;13:445
*Spermophilus alaschanicus*	*SSU rRNA*	*Cryptosporidium* sp. Chipmunk genotype V	PQ568971	*Cryptosporidium* sp. Chipmunk genotype V 808/815 (99.14%) MZ478133	Xu J, Liu H, Jiang Y, Jing H, Cao J, Yin J, et al. Genotyping and subtyping of *Cryptosporidium* spp. and *Giardia duodenalis* isolates from two wild rodent species in Gansu Province, China. *Sci Rep*. 2022;12:12,178
*Spermophilus undulatus*	*SSU rRNA*	*Cryptosporidium* sp. Chipmunk genotype V	PQ569066	*Cryptosporidium* sp. Chipmunk genotype V 802/812(98.77%) MW521250	Feng K, Yang S, Xu Y, Wen L, Chen J, Zhang W, et al. Molecular characterization of *Cryptosporidium* spp., *Giardia* spp. and *Enterocytozoon bieneusi* in eleven wild rodent species in China: common distribution, extensive genetic diversity and high zoonotic potential. *One Health*. 2024;18:100,750
*Spermophilus undulatus*	*SSU rRNA*	*Cryptosporidium rubeyi*	PQ569065	*Cryptosporidium rubeyi* 817/832(98.20%) DQ295014	Pereira Md, Li X, McCowan B, Phillips RL, Atwill ER. Multiple unique *Cryptosporidium* isolates from three species of ground squirrels (*Spermophilus beecheyi*, *S*. *beldingi*, and *S*. *lateralis*) in California. *Appl Environ Microbiol*. 2010;76:8269–76 Li X, Pereira Md, Larsen R, Xiao C, Phillips R, Striby K, et al. *Cryptosporidium rubeyi* n. sp. (Apicomplexa: Cryptosporidiidae) in multiple *Spermophilus* ground squirrel species. *Int J Parasitol Parasites Wildl*. 2015;4:343–50
*Spermophilus undulatus*	*SSU rRNA*	*Cryptosporidium* sp.	PX116793	*Cryptosporidium* sp. 810/813 (99.63%) MN599025	García-Livia K, Martín-Alonso A, Foronda P. Diversity of *Cryptosporidium* spp. in wild rodents from the Canary Islands, Spain. *Parasit Vectors*. 2020;13:445
*Spermophilus erythrogenys*	*SSU rRNA*	*Cryptosporidium* sp. Chipmunk genotype V	PQ569074	*Cryptosporidium* sp. Chipmunk genotype V 787/796 (98.87%) MZ478133	Xu J, Liu H, Jiang Y, Jing H, Cao J, Yin J, et al. Genotyping and subtyping of *Cryptosporidium* spp. and *Giardia duodenalis* isolates from two wild rodent species in Gansu Province, China. *Sci Rep*. 2022;12:12,178
*Spermophilus erythrogenys*	*SSU rRNA*	*Cryptosporidium rubeyi*	PQ569073	*Cryptosporidium rubeyi* 799/814 (98.16%) AY462233	Atwill ER, Phillips R, Pereira MD, Li X, McCowan B. Seasonal shedding of multiple *Cryptosporidium* genotypes in California ground squirrels (*Spermophilus beecheyi*). *Appl Environ Microbiol*. 2004;70:6748–52 Li X, Pereira Md, Larsen R, Xiao C, Phillips R, Striby K, et al. *Cryptosporidium rubeyi* n. sp. (Apicomplexa: Cryptosporidiidae) in multiple *Spermophilus* ground squirrel species. *Int J Parasitol Parasites Wildl*. 2015;4:343–50
*Rhombomys opimus*	*SSU rRNA*	*Cryptosporidium ubiquitum*	PQ569077	*Cryptosporidium ubiquitum* 800/800 (100%) MW521251	Chen J, Wang W, Lin Y, Sun L, Li N, Guo Y, et al. Genetic characterizations of *Cryptosporidium* spp. from pet rodents indicate high zoonotic potential of pathogens from chinchillas. *One Health*. 2021;13:100,269
*Rhombomys opimus*	*SSU rRNA*	*Cryptosporidium* sp. Vole genotype III	PQ569076	*Cryptosporidium* sp. Vole genotype III 784/791(99.12%) MH145329	Horčičková M, Čondlová Š, Holubová N, Sak B, Květoňová D, Hlásková L, et al. Diversity of *Cryptosporidium* in common voles and description of *Cryptosporidium alticolis* sp. n. and *Cryptosporidium microti* sp. n. (Apicomplexa: Cryptosporidiidae). *Parasitology*. 2019;146:220–233
*Meriones* *libycus*	*SSU rRNA*	*Cryptosporidium ubiquitum*	PX116794	*Cryptosporidium ubiquitum* 823/823 (100%) MW521251	Chen J, Wang W, Lin Y, Sun L, Li N, Guo Y, et al. Genetic characterizations of *Cryptosporidium* spp. from pet rodents indicate high zoonotic potential of pathogens from chinchillas. *One Health*. 2021;13:100,269
*Mus* *musculus*	*SSU rRNA*	*Cryptosporidium* sp.	PQ569064	*Cryptosporidium* sp. 798/801 (99.63%) MN599025	García-Livia K, Martín-Alonso A, Foronda P. Diversity of *Cryptosporidium* spp. in wild rodents from the Canary Islands, Spain. *Parasit Vectors*. 2020;13:445
*Ellobius* *talpinus*	*SSU rRNA*	*Cryptosporidium* sp. Vole genotype III	PQ569069	*Cryptosporidium* sp. Vole genotype III 741/750(98.80%) MH145329	Horčičková M, Čondlová Š, Holubová N, Sak B, Květoňová D, Hlásková L, et al. Diversity of *Cryptosporidium* in common voles and description of *Cryptosporidium alticolis* sp. n. and *Cryptosporidium microti* sp. n. (Apicomplexa: Cryptosporidiidae). *Parasitology*. 2019;146:220–233
*Ellobius* *talpinus*	*SSU rRNA*	*Cryptosporidium ubiquitum*	PQ569068	*Cryptosporidium ubiquitum* 802/809 (99.13%) MW521251	Chen J, Wang W, Lin Y, Sun L, Li N, Guo Y, et al. Genetic characterizations of *Cryptosporidium* spp. from pet rodents indicate high zoonotic potential of pathogens from chinchillas. *One Health*. 2021;13:100,269
*Ondatra* *zibethicus*	*SSU rRNA*	*Cryptosporidium* sp. Muskrat genotype I	PQ569079	*Cryptosporidium* sp. Muskrat genotype I 805/811 (99.26%) EF641013	Feng Y, Alderisio KA, Yang W, Blancero LA, Kuhne WG, Nadareski CA, et al. *Cryptosporidium* genotypes in wildlife from a new york watershed. *Appl Environ Microbiol*. 2007;73:6475–83
*Microtus* *obscurus*	*SSU rRNA*	*Cryptosporidium* sp. Vole genotype V	PQ569075	*Cryptosporidium* sp. Vole genotype V 806/813 (99.14%) PP177946	Feng K, Yang S, Xu Y, Wen L, Chen J, Zhang W, et al. Molecular characterization of *Cryptosporidium* spp., *Giardia* spp. and *Enterocytozoon bieneusi* in eleven wild rodent species in China: Common distribution, extensive genetic diversity and high zoonotic potential. *One Health*. 2024;18:100,750
*Ochotona* *pallasi*	*SSU rRNA*	*Cryptosporidium* sp. Mongolian pika genotype	PV794561	*Cryptosporidium* sp. 781/819 (95.36%) PP177944	Feng K, Yang S, Xu Y, Wen L, Chen J, Zhang W, et al. Molecular characterization of *Cryptosporidium* spp., *Giardia* spp. and *Enterocytozoon bieneusi* in eleven wild rodent species in China: Common distribution, extensive genetic diversity and high zoonotic potential. *One Health*. 2024;18:100,750
*Ochotona* *pallasi*	*SSU rRNA*	*Cryptosporidium* sp. Yak genotype	OR557401	*Cryptosporidium* sp. Yak genotype 773/787(98.22%) KF971356	Ma J, Cai J, Ma J, Feng Y, Xiao L. Occurrence and molecular characterization of *Cryptosporidium* spp. in yaks (*Bos grunniens*) in China. *Vet Parasitol*. 2014;202:113–8
*Ochotona* *pallasi*	*SSU rRNA*	*Cryptosporidium ryanae*	OR557411	*Cryptosporidium ryanae* 821/823 (99.76%) KP793013	Qi M, Wang H, Jing B, Wang D, Wang R, Zhang L. Occurrence and molecular identification of *Cryptosporidium* spp. in dairy calves in Xinjiang, Northwestern China. *Vet Parasitol*. 2015;212:404–7
*Equus* *asinus*	*SSU rRNA*	*Cryptosporidium equi*	PV794554	*Cryptosporidium equi* 740/743 (99.60%) ON384432	Xu J, Liu H, Jiang Y, Jing H, Cao J, Yin J, et al. Genotyping and subtyping of *Cryptosporidium* spp. and *Giardia duodenalis* isolates from two wild rodent species in Gansu Province, China. *Sci Rep*. 2022;12:12,178
*Equus* *asinus*	*SSU rRNA*	*Cryptosporidium hominis*	PV794553	*Cryptosporidium hominis* 776/789 (98.35%) KR296812	Lebbad M, Winiecka-Krusnell J, Insulander M, Beser J. Molecular characterization and epidemiological investigation of *Cryptosporidium hominis* IkA18G1 and *C*. *hominis* monkey genotype IiA17, two unusual subtypes diagnosed in Swedish patients. *Exp Parasitol*. 2018;188:50–57
*Pipistrellus pipistrellus*	*SSU rRNA*	*Cryptosporidium muris*	PV794556	*Cryptosporidium muris* 768/772 (99.48%) EU245043	Kvác M, Sak B, Kvetonová D, Ditrich O, Hofmannová L, Modrý D, et al. Infectivity, pathogenicity, and genetic characteristics of mammalian gastric *Cryptosporidium* spp. in domestic ruminants. *Vet Parasitol*. 2008;153:363–7
*Pipistrellus pipistrellus*	*SSU rRNA*	*Cryptosporidium* sp. Bat genotype IV	PV794555	*Cryptosporidium* sp. Bat genotype IV 782/788 (99.24%) KR819168	Kváč M, Hořická A, Sak B, Prediger J, Salát J, Širmarová J, et al. Novel *Cryptosporidium* bat genotypes III and IV in bats from the USA and Czech Republic. *Parasitol Res*. 2015;114:3917–21
*Cervus* *nippon*	*SSU rRNA*	*Cryptosporidium* sp. Deer genotype	PV794558	*Cryptosporidium* sp. Deer genotype 813/820 (99.15%) MN056201	Tao WF, Ni HB, Du HF, Jiang J, Li J, Qiu HY, et al. Molecular detection of *Cryptosporidium* and *Enterocytozoon bieneusi* in dairy calves and sika deer in four provinces in Northern China. *Parasitol Res*. 2020;119:105–114
*Vulpes* *vulpes*	*SSU rRNA*	*Cryptosporidium canis*	PV794562	*Cryptosporidium canis* 810/818 (99.02%) AB210854	Satoh M, Matsubara-Nihei Y, Sasaki T, Nakai Y. Characterization of *Cryptosporidium canis* isolated in Japan. *Parasitol Res*. 2006;99:746–8
*Panthera* *tigris*	*SSU rRNA*	*Cryptosporidium felis*	PV794557	*Cryptosporidium felis* 712/724 (98.34%) DQ836340	Santín M, Trout JM, Vecino JA, Dubey JP, Fayer R. *Cryptosporidium*, *Giardia* and *Enterocytozoon bieneusi* in cats from Bogota (Colombia) and genotyping of isolates. *Vet Parasitol*. 2006;141:334–9
*Anser* *anser*	*SSU rRNA*	*Cryptosporidium proventriculi*	PV794559	*Cryptosporidium proventriculi* 790/796 (99.25%) MW783465	Liao C, Wang T, Koehler AV, Hu M, Gasser RB. *Cryptosporidium* of birds in pet markets in Wuhan city, Hubei, China. *Curr Res Parasitol Vector Borne Dis*. 2021;1:100,025
*Larus* *ichthyaetus*	*SSU rRNA*	*Cryptosporidium* sp. Goose genotype I	PV794560	*Cryptosporidium* sp. Goose genotype I 667/667(99.70%) AY120912	Xiao L, Sulaiman IM, Ryan UM, Zhou L, Atwill ER, Tischler ML, et al. Host adaptation and host-parasite co-evolution in *Cryptosporidium*: implications for taxonomy and public health. *Int J Parasitol*. 2002;32:1773–85
*Larus* *fuscus*	*SSU rRNA*	*Cryptosporidium* sp. Goose genotype I	PX248577	*Cryptosporidium* sp. Goose genotype I 668/669 (99.85%) AY504516	Zhou L, Kassa H, Tischler ML, Xiao L. Host-adapted *Cryptosporidium* spp. in Canada geese (*Branta canadensis*). *Appl Environ Microbiol*. 2004;70:4211–5
*Equus* *asinus*	GP60	*Cryptosporidium hominis*	PV800351	*Cryptosporidium hominis* 753/753 (100%) KU727290	Lebbad M, Winiecka-Krusnell J, Insulander M, Beser J. Molecular characterization and epidemiological investigation of *Cryptosporidium hominis* IkA18G1 and *C*. *hominis* monkey genotype IiA17, two unusual subtypes diagnosed in Swedish patients. *Exp Parasitol*. 2018;188:50–57
*Rhombomys opimus*	GP60	*Cryptosporidium ubiquitum*	PQ588454	*Cryptosporidium ubiquitum* 790/825 (95.76%) KT027488	Stenger BLS, Clark ME, Kváč M, Khan E, Giddings CW, Prediger J, et al. North American tree squirrels and ground squirrels with overlapping ranges host different *Cryptosporidium* species and genotypes. *Infect Genet Evol*. 2015;36:287–293
*Meriones* *libycus*	GP60	*Cryptosporidium ubiquitum*	PQ606087	*Cryptosporidium ubiquitum* 790/825 (95.76%) KT027488	Stenger BLS, Clark ME, Kváč M, Khan E, Giddings CW, Prediger J, et al. North American tree squirrels and ground squirrels with overlapping ranges host different *Cryptosporidium* species and genotypes. *Infect Genet Evol*. 2015;36:287–293
*Meriones* *libycus*	GP60	*Cryptosporidium ubiquitum*	PQ606086	*Cryptosporidium ubiquitum* 776/822 (94.40%) KT027488	Stenger BLS, Clark ME, Kváč M, Khan E, Giddings CW, Prediger J, et al. North American tree squirrels and ground squirrels with overlapping ranges host different *Cryptosporidium* species and genotypes. *Infect Genet Evol*. 2015;36:287–293

In addition, a novel *Cryptosporidium* genotype (named here as *Cryptosporidium* Mongolian pika genotype) was successfully amplified from Mongolian pikas (*n* = 4). This genotype shared 95.36% *SSU rRNA* gene sequence identity with *Cryptosporidium* sp. (PP177944) reported in South China field mouse (*Apodemus draco*) in China. In the phylogenetic tree, *Cryptosporidium* Mongolian pika genotype belonged to an independent clade, which was a sister group to *Cryptosporidium* muskrat genotype I and vole genotype V. Interestingly, Mongolia pikas were additionally infected with *Cryptosporidium* yak genotype and *C. ryanae*. Ecologically, habitats of the Mongolia pika overlap with multiple Bovidae, such as Przewalski’s gazelle (*Procapra przewalskii*), Siberian ibex (*Capra ibex*), and cattle.

Furthermore, the *GP60* gene was targeted for subtyping *C. hominis* and *C. ubiquitum*. The *C*. *ubiquitum* identified in this study belonged to subtype *C*. *ubiquitum* XIIb, while *C*. *hominis* belonged to subtype *C*. *hominis* IkA18G1 (Additional File 3).

## Discussion

*Cryptosporidium* infection poses health threats to humans, livestock, pets, and wildlife. In this study, a broad-spectrum surveillance of *Cryptosporidium* species was carried out, involving 273 imported donkeys, 1224 wild-living mammals, 124 reptiles, and 234 birds, which represented 75 animal species. From these hosts, 19 *Cryptosporidium* species and genotypes, including a novel one, were demonstrated. The highest genetic diversity was observed in rodents, which harbored seven species or genotypes. Notably, this study showed for the first time the occurrence of *Cryptosporidium* in red-cheeked ground squirrels (*Spermophilus erythrogenys*), great gerbils (*Rhombomys opimus*), northern mole voles (*Ellobius tanceri*), Libyan jirds (*Meriones libycus*), and Mongolian pikas.

Host preference of *Cryptosporidium*, as exemplified by *Cryptosporidium bovis* in Bovidae, *Cryptosporidium xiaoi* in Caprinae, and *Cryptosporidium galli* in birds, was already reported [24–26]. In this study, *C*. *canis* was detected in red fox, *C*. *felis* in zoo tiger, *C*. *proventriculi* in greylag goose (*Anser anser*), *C*. *equi* in imported donkeys, *Cryptosporidium* deer genotype in sika deer, bat genotype IV in common pipistrelle (*Pipistrellus pipistrellus*), vole genotype V in Altai voles (*Microtus obscurus*), muskrat genotype I in muskrats (*Ondatra zibethicus*), vole genotype III in northern mole voles (*Ellobius talpinus*) and great gerbils, chipmunk genotype V in Himalayan marmots (*Marmota himalayana*), and goose genotype I in Pallas’s gull (*Larus ichthyaetus*). These findings also suggest that in northern China, some *Cryptosporidium* species and genotypes have preference for certain host taxa, both among mammals and birds.

Rodents (Mammalia: Rodentia), with more than 2200 species globally, are considered a major threat to public health as they can act as reservoirs or carriers of several pathogenic microorganisms, including *Cryptosporidium* [27]. *Cryptosporidium rubeyi* was described for the first time in California ground squirrels (*Spermophilus beecheyi*) [28], subsequently also detected in Belding’s ground squirrels (*Spermophilus beldingi*) and golden-mantled ground squirrels (*Spermophilus lateralis*) [29]. In this study, *C*. *rubeyi* was identified for the first time in long-tailed ground squirrel (*Spermophilus undulatus*) and red-cheeked ground squirrel, showing a prevalence of 11.68% (25/124). *Cryptosporidium* chipmunk genotype V, previously identified in Himalayan marmots with a prevalence of 0.25% (1/399) [30], was also detected here in Himalayan marmots (6.25%, 6/96), as well as Alashan ground squirrels (18.18%, 2/11), long-tailed ground squirrels (5.53%, 11/199) and red-cheeked ground squirrels (5.22%, 6/115). Our findings suggest that *C*. *rubeyi* and *Cryptosporidium* chipmunk genotype V are host-specific for local members of Sciuridae.

The third rodent-associated species, *C*. *ubiquitum*, is a globally recognized zoonotic protozoon. It has the widest host range among all *Cryptosporidium* species, including domestic and wild ruminants, omnivores, primates, and rodents [9, 31]. Previously, *C*. *ubiquitum* was detected in Eastern gray squirrel (*Sciurus carolinensis*) in Italy, in American red squirrel (*Tamiasciurus hudsonicus*) and fox squirrel (*Sciurus niger*) in North America, as well as in Pallas’s squirrel (*Callosciurus erythraeus*) and South China field mouse (*A. draco*) in China [32, 33]. In this study, *C*. *ubiquitum* was detected for the first time in Muridae, such as great gerbils, Libyan jirds, and northern mole voles. Furthermore, the phylogenetic tree based on the *GP60* gene showed that the sequences of *C*. *ubiquitum* stains (representing *C*. *ubiquitum* XIIb subtype) from great gerbils and Libyan jirds clustered together with the strains from humans, gray squirrels, sheep, and the environment (Additional File 4). The great gerbil is the most dominant rodent species in desert and semi-desert regions of XUAR, IMAR, and Central Asia [34]. Therefore, this finding suggests that the rodent-derived *C*. *ubiquitum* XIIb subtype may spill over to humans, especially in densely populated rural landscapes that overlap with regions inhabited by the great gerbil.

In addition, a notable finding in this study was 17 positive samples identified as *Cryptosporidium* sp. in wild rodents (Himalayan marmots, gray marmots, long-tailed ground squirrels, and house mice), and their corresponding sequences (PX116791, PX116792, PX116793, and PQ569064) exhibited over 99% similarities to the reference sequence (MN599025, *Cryptosporidium* sp. isolated from house mice in Spain). Phylogenetically, these clustered in a single clade (Fig. 2). Moreover, we noted that this species exhibited 98.66% similarity to *C*. *occultus* (MW090932) and formed a closely related branch in the phylogenetic tree (Additional File 4), suggesting that it may represent a variant of *C*. *occultus*. Notably, the first three hosts in the phylogenetic tree, belonging to Sciuridae, typically inhabit alpine meadows. By contrast, house mice, belonging to Muridae, are predominantly found in human settlements. This result suggests that this unnamed *Cryptosporidium* sp. has the potential of cross-species transmission between Sciuridae and Muridae.

Bats, the second most diverse mammalian order after Rodentia, have garnered significant attention as natural reservoirs of numerous pathogens [35]. *Cryptosporidium* bat genotype IV was reported for the first time in 2015 in common pipistrelle (*P. pipistrellus*) from the Czech Republic and later detected in the straw-colored fruit bat (*Eidolon helvum*) in Nigeria [36, 37]. Another species, *C*. *muris* can infect a wider range of hosts, including humans, various rodents, artiodactyls, carnivores, and other mammals, as well as birds such as ostriches (*Struthio camelus*) [38–40]. In our study, both *Cryptosporidium* bat genotype IV and* C*. *muris* were detected in common pipistrelle individuals. These findings present the first evidence for *Cryptosporidium* bat genotype IV in China and for the occurrence of *C*. *muris* in bats, suggesting that (1) *Cryptosporidium* bat genotype IV occurs in a larger geographical region, and (2) *C*. *muris* has an even broader host range compared with data in previous reports. Given the zoonotic potential of *Cryptosporidium* bat genotype IV and *C*. *muris*, the risk of their transmission between bats and humans should receive more attention in the future.

Pikas (Lagomorpha: Ochotonidae) are small, rabbit-like mammals, which belong to the genus *Ochotona*, including 30 species worldwide [41]. Among these, the Mongolian pika (*Ochotona pallasi*) is a unique species, currently confined to plateau-steppe and talus habitats in a small area of China, Mongolia, and Russia [42]. Previously, only *Yersinia pestis* and a novel trypanosomatid species were reported in Mongolian pikas [43, 44]. In this study, we detected *C*. *ryanae*, *Cryptosporidium* yak genotype and a novel *Cryptosporidium* genotype in these hosts. Considering the first and second species, the *Cryptosporidium* yak genotype has been reported in yaks in China [45], while *C*. *ryanae* was identified in water buffaloes (*Bubalus bubalis*), cattle, cats, red deer (*Cervus elaphus*), lowland pacas (*Cuniculus paca*), marsh deer (*Blastocerus dichotomus*), and humans [46–52]. Reports of *C*. *ryanae* in multiple hosts attest that this species lacks strict host specificity to bovids. Notably, Mongolian pikas share extensive, overlapping habitats with local livestock, including yaks and cattle and other bovids, suggesting potential cross-species transmission via environmental contamination (e.g., water sources contaminated with oocysts shed by infected bovids).

The third species detected in Mongolian pikas in this study was provisionally named here as *Cryptosporidium* Mongolian pika genotype and shared only 95.66% (771/806) *SSU rRNA* sequence identity with the closest clade represented by *Cryptosporidium* sp. (PP177944) reported in South China field mouse in China. The phylogenetic analysis based on the small subunit ribosomal RNA (*SSU rRNA*) gene showed that *Cryptosporidium* Mongolian pika genotype clustered with *Cryptosporidium* muskrat genotype I and vole genotype V. Although *Cryptosporidium* has been previously reported in plateau pikas, the phylogenetic tree shows that the sequence (KX949423) forms an independent branch and exhibits differences in the *Cryptosporidium* Mongolian pika genotype (Additional File 4). These findings further underscore the potential for cross-species transmission in the case of certain *Cryptosporidium* species. While the zoonotic capacity and pathogenicity of the newly identified Mongolian pika genotype remain unclear, this study provides critical epidemiological insights into *Cryptosporidium* infections in pikas and highlights the need for expanded surveillance at wildlife–livestock interfaces.

Transmission of *Cryptosporidium* species across borders has been well documented through multiple pathways, including human activities, contaminated water sources, and animal trade, with globalization substantially amplifying dissemination risks [53, 54]. In this investigation, we conducted *Cryptosporidium* surveillance on 273 free-ranging donkeys imported from Kyrgyzstan and 234 wild birds. *Cryptosporidium hominis* and *Cryptosporidium* *equi* were detected in imported donkeys. In addition, XUAR lies at the convergence of three major avian migratory flyways: the West Asian–East African Flyway, the Central Asian–South Asian Flyway, and the East Asian–Australasian Flyway [55, 56]. In this study, *C.* *proventriculi* was identified in greylag geese (*Anser anser*), and *Cryptosporidium* goose genotype I was found in gull species (e.g., *Larus ichthyaetus*). These results support the significant role of imported equids and migratory birds in the transboundary transmission of parasites.

However, ecological factors serve as potential drivers for *Cryptosporidium* transmission dynamics. For instance, global warming-induced alterations in migratory bird routes and timing may lead to increased interactions between wildlife and human/livestock populations, thereby elevating pathogen spillover risks [57, 58]. Furthermore, research on *Cryptosporidium* prediction with climate change scenarios has indicated the geographic expansion of suitable habitats in northern China [59]. In this study, we detected 19 *Cryptosporidium* species in 75 host species in northern China. Combined with these findings, assessing the public health risks of *Cryptosporidium* in northern China has become both urgent and important.

## Conclusions

This study investigated *Cryptosporidium* infections in 1855 wildlife animals (belonging to 75 species) across 20 counties/cities in XUAR and IMAR, northern China. A total of 18 known *Cryptosporidium* species/genotypes were identified, with the first documented detection of *Cryptosporidium* in red-cheeked ground squirrels, great gerbils, northern mole voles, Libyan jirds, and Mongolian pikas. These results expand the known host range and geographic distribution of *Cryptosporidium* species. In addition, a novel *Cryptosporidium* genotype was identified in Mongolian pikas. More importantly, the detection of *C*. *bovis* and the yak genotype in Mongolian pikas, alongside *C*. *muris* in common pipistrelle bat, suggest potential cross-species transmission of *Cryptosporidium* among sympatric species due to overlapping ecological niches.

## Supplementary Information

Additional file 1. Table S1. The sources of the samples in this study and the prevalence of *Cryptosporidium* species/genotypes in them.

Additional file 2. Table S2. The data of (A) primers and (B) cycling conditions used in this study. The genetic markers that served for identification of species were as follows: *CytB *for rodent species; *16SrRNA* for bird species; D-loop mtDNA for bird feces; *COX1* for reptile species; *SSU rRNA* and *GP60 *for *Cryptosporidium *spp.

Additional file 3. Figure S1. Phylogenetic tree of *Cryptosporidium ubiquitum* and *Cryptosporidium hominis* was conducted using the maximum-likelihood method based on *GP60* gene sequences under the K2+I model with 1,000 bootstrap replicates. Novel sequences identified in this study are denoted by black circles and labeled with GenBank accession numbers, host species and country of origin. 

Additional file 4. Figure S2. Phylogenetic tree of *Cryptosporidium* was conducted using the maximum-likelihood method based on *SSUrRNA* gene sequences under the T92+I model with 1,000 bootstrap replicates.

## Data Availability

The sequences obtained and analyzed during the present study are deposited in the GenBank database under the accession nos. *16S rRNA*: PQ394778–PQ394785, PQ451723–PQ451727, and PQ459365; *COX1*: PQ451734–PQ451736; D-loop mtDNA: PQ373942–PQ373943, PQ393132–PQ393138, and PQ474771; *CytB*: PQ450150–PQ450159, PQ450161–PQ450162, PQ450165, PQ450168, PQ474772–PQ474773, and PQ581939; *Cryptosporidium SSU rRNA*: PQ568971.2, PQ569063–PQ569074, PQ569075.2–PQ569083, PQ865466, PQ865468–PQ865469, PV794553.2–PV794558, PV794559.2–PV794562, PX116791–PX116794, and PX248577; *C. ubiquitum GP60*: PQ588454 and PQ606086–PQ606087; *C. hominis GP60*: PV800351.

## Abbreviations

XUAR: Xinjiang Uygur Autonomous Region
IMAR: Inner Mongolia Autonomous Region
*SSU rRNA*: Small subunit ribosomal RNA
*GP60*: 60 KDa glycoprotein
*16S** rRNA*: 16S ribosomal RNA
*COX1*: Cytochrome coxidase subunit I
*Cytb*: Cytochrome B
D-loop mtDNA: Displacement loop region mitochondrial DNA
