# Supplementary material for: Investigation of Cryptosporidium infection in a broad range of hosts in northern China
Source: Parasit Vectors. 2025 Nov 26;18:509. doi: 10.1186/s13071-025-07152-9 (PMC12750556; doi:10.1186/s13071-025-07152-9)
Supplement: Supplementary file 1 — Additional file 1. Table S1. The sources of the samples in this study and the prevalence of Cryptosporidium species/genotypes in them. [file 13071_2025_7152_MOESM1_ESM.docx]

**Additional file1: Table S1.** The source of the samples in this study and the prevalence of *Cryptosporidium* species/genotypes in them.

| **Animal** | **Sample type** | **No. examined** | **No. Positive**  **(%)** | **Species / genotypes (No. of positive samples)** | **Genbank number** | **Sampling site**  **(No. of samples)** | **Collection time**  **(year)** | **Permit** |
| --- | --- | --- | --- | --- | --- | --- | --- | --- |
| Mammalian | | | | | | | | |
| Rodentia | | | | | | | | |
| *Marmota himalayana* | small intestine | 96 | 6  (6.25) | *Cryptosporidium* sp.  (n=3)  *Cryptosporidium* chipmunk genotype V  (n=3) | PX116791  PQ569063 | Jinghe County  (n=3)  Tumushuk City  (n=3)  Pishan County  (n=90) | 2021, 2023 | Approval No. A2018-143-01  Approval No. A2022-029-01 |
| *Marmota baibacina* | small intestine | 2 | 2  (100) | *Cryptosporidium* sp.  (n=2) | PX116792 | Jinghe County  (n=2) | 2021 | Approval No. A2018-143-01 |
| *Spermophilus alaschanicus* | small intestine | 11 | 2  (18.18) | *Cryptosporidium* chipmunk genotype V  (n=2) | PQ568971 | Wuchuan County  (n=11) | 2023 | Approval No. A2022-029-01 |
| *Spermophilus undulatus* | small intestine | 199 | 35  (17.59) | *Cryptosporidium* chipmunk genotype V  (n=11)  *Cryptosporidium rubeyi*  (n=14)  *Cryptosporidium* sp.  (n=10) | PQ569066  PQ569065  PX116793 | Wenquan County  (n=41)  Jinghe County  (n=102)  Wusu City  (n=56) | 2022, 2023 | Approval No. A2022-029-01 |
| *Spermophilus erythrogenys* | small intestine | 115 | 17  (14.78) | *Cryptosporidium* chipmunk genotype V  (n=6)  *Cryptosporidium rubeyi*  (n=11) | PQ569074  PQ569073 | Wenquan County  (n=27)  Emin County  (n=8)  Tuoli County  (n=36)  Bole City  (n=44) | 2022, 2023 | Approval No. A2018-143-01  Approval No. A2022-029-01 |
| *Tamias*  *sibiricus* | small intestine | 1 | 0 | / | / | Fuhai County  (n=1) | 2023 | Approval No. A2022-029-01 |
| *Rhombomys opimus* | small intestine | 146 | 8  (5.48) | *Cryptosporidium ubiquitum*  (n=5)  *Cryptosporidium* vole genotype III  (n=3) | PQ569077  PQ569076 | Karamay City  (n=52)  Shihezi City  (n=20)  Manasi County  (n=52)  Qitai Countty  (n=12)  Bayan Nur City  (n=10) | 2020, 2023 | Approval No. A2018-143-01  Approval No. A2022-029-01 |
| *Meriones meridianus* | small intestine | 5 | 0 | / | / | Wusu City  (n=5) | 2021 | Approval No. A2018-143-01 |
| *Meriones*  *libycus* | small intestine | 122 | 14  (11.48) | *Cryptosporidium ubiquitum*  (n=14) | PX116794 | Wusu City  (n=122) | 2023 | Approval No. A2022-029-01 |
| *Meriones tamariscinus* | small intestine | 8 | 0 | / | / | Wusu City  (n=8) | 2023 | Approval No. A2022-029-01 |
| *Mus*  *musculus* | small intestine | 7 | 2  (28.57) | *Cryptosporidium* sp.  (n=2) | PQ569064 | Fuhai County  (n=6)  Tumushuk City  (n=1) | 2023 | Approval No. A2022-029-01 |
| *Rattus*  *norvegicus* | small intestine | 15 | 0 | / | / | Shihezi City  (n=15) | 2023 | Approval No. A2022-029-01 |
| *Ellobius*  *talpinus* | small intestine | 13 | 2  (15.38) | *Cryptosporidium* vole genotype III  (n=1)  *Cryptosporidium ubiquitum*  (n=1) | PQ669069  PQ569068 | Wujiaqu City  (n=2)  Fuhai County  (n=11) | 2019 | Approval No. A2018-143-01 |
| *Ondatra*  *zibethicus* | small intestine | 5 | 3  (60) | *Cryptosporidium* muskrat genotype I  (n=3) | PQ569079 | Makit County  (n=5) | 2022 | Approval No. A2018-143-01 |
| *Microtus*  *obscurus* | small intestine | 19 | 7  (36.84) | *Cryptosporidium* vole genotype V  (n=7) | PQ569075 | Nilka County  (n=19) | 2015 | Approval No. AECSU2014-03 |
| *Allactaga*  *elater* | small intestine | 29 | 0 | / | / | Manasi County  (n=1)  Qitai County  (n=28) | 2021, 2025 | Approval No. A2018-143-01  Approval No. A2022-029-01 |
| *Allactaga*  *sibirica* | small intestine | 3 | 0 | / | / | Qitai County  (n=3) | 2025 | Approval No. A2022-029-01 |
| *Dipus*  *sagitta* | small intestine | 1 | 0 | / | / | Manasi County  (n=1) | 2023 | Approval No. A2022-029-01 |
| Lagomorpha | | | | | | | | |
| *Ochotona*  *pallasi* | small intestine | 81 | 12  (14.81) | *Cryptosporidium* Mongolian Pika genotype  (n=4)  *Cryptosporidium* yak genptype  (n=6)  *Cryptosporidium ryanae*  (n=2) | PV794561  OR557400  OR557411 | Qitai County  (n=81) | 2021, 2023 | Approval No. A2018-143-01  Approval No. A2022-029-01 |
| *Lepus*  *yarkandensis* | small intestine | 2 | 0 | / | / | Tumushuk City  (n=2) | 2023 | Approval No. A2022-029-01 |
| Soricomorpha | | | | | | | | |
| *Sorex*  *araneus* | small intestine | 2 | 0 | / | / | Shihezi City  (n=2) | 2023 | Approval No. A2022-029-01 |
| Perissodactyla | | | | | | | | |
| *Equus*  *asinus* | small intestine | 273 | 13  (4.76) | *Cryptosporidium equi*  (n=7)  *Cryptosporidium hominis*  (n=6) | PV794554  PV794553 | Uqia County  (n=273) | 2023, 2024 | Approval No. A2022-029-01 |
| Chiroptera | | | | | | | | |
| *Pipistrellus pipistrellus* | small intestine | 289 | 15  (5.19) | *Cryptosporidium muris*  (n=3)  *Cryptosporidium*  bat genotype IV  (n=12) | PV794556  PV794555 | Nilka City  (n=289) | 2017, 2019 | Approval No. AECSUKJ2015−  01  Approval No. A2018-143-01 |
| Artiodactyla | | | | | | | | |
| *Sus*  *scrofa* | small intestine | 4 | 0 | / | / | Bachu County  (n=4) | 2024 | Approval No. A2022-029-01 |
| *Procapra*  *przewalskii* | small intestine | 3 | 0 | / | / | Wusu City  (n=3) | 2024 | Approval No. A2022-029-01 |
| *Cervus*  *nippon* | faeces | 3 | 1  (33.33) | *Cryptosporidium*  deer genotype  (n=1) | PV794558 | Shihezi City  (n=3) | 2024 | Approval No. A2022-029-01 |
| *Camelus*  *bactrianus* | faeces | 1 | 0 | / | / | Shihezi City  (n=1) | 2024 | Approval No. A2022-029-01 |
| Carnivora | | | | | | | | |
| *Vulpes*  *vulpes* | small intestine | 16 | 2  (12.5) | *Cryptosporidium canis*  (n=2) | PV794562 | Nilka County  (n=16) | 2015-2024 | Approval No. AECSU2013-18  Approval No. AECSUKJ2015−  01  Approval No. A2018-143-01  Approval No. A2022-029-01 |
| *Vormela*  *peregusna* | small intestine | 8 | 0 | / | / | Jinghe County  (n=6)  Karamay City  (n=2) | 2014-2024 | Approval No. AECSU2013-18  Approval No. AECSUKJ2015−  01  Approval No. A2018-143-01  Approval No. A2022-029-01 |
| *Meles*  *meles* | small intestine | 6 | 0 | / | / | Nilka County  (n=6) | 2018 | Approval No. AECSUKJ2015−  01 |
| *Lynx*  *lynx* | small intestine | 3 | 0 | / | / | Emin County  (n=3) | 2019 | Approval No. A2018-143-01 |
| *Canis*  *lupus* | faeces | 1 | 0 | / | / | Shihezi City  (n=1) | 2024 | Approval No. A2022-029-01 |
| *Panthera*  *leo* | faeces | 2 | 0 | / | / | Shihezi City  (n=2) | 2024 | Approval No. A2022-029-01 |
| *Ursus*  *arctos* | faeces | 1 | 0 | / | / | Shihezi City  (n=1) | 2024 | Approval No. A2022-029-01 |
| *Ursus*  *thibetanus* | faeces | 1 | 0 | / | / | Shihezi City  (n=1) | 2024 | Approval No. A2022-029-01 |
| *Panthera*  *tigris* | faeces | 1 | 1  (100) | *Cryptosporidium felis*  (n=1) | PV794557 | Shihezi City  (n=1) | 2024 | Approval No. A2022-029-01 |
| Erinaceomorpha | | | | | | | | |
| *Erinaceus* | small intestine | 5 | 0 | / | / | Bayan Nur City  (n=5) | 2023 | Approval No. A2022-029-01 |
| Primates | | | | | | | | |
| *Cercopithecidae* | small intestine | 1 | 0 | / | / | Shihezi City  (n=1) | 2024 | Approval No. A2022-029-01 |
| Reptilian | | | | | | | | |
| Lacertidae | | | | | | | | |
| *Eremias*  *velox* | small intestine | 40 | 0 | / | / | Wusu City  (n=24)  Bachu County  (n=16) | 2022, 2023 | Approval No. A2018-143-01  Approval No. A2022-029-01 |
| Agamidae | | | | | | | | |
| *Phrynocephalus versicolor* | small intestine | 20 | 0 | / | / | Wusu City  (n=20) | 2023 | Approval No. A2022-029-01 |
| *Phrynocephalus guttatus* | small intestine | 24 | 0 | / | / | Wusu City  (n=24) | 2023 | Approval No. A2022-029-01 |
| *Laudakia* | | | | | | | | |
| *Paralaudakia lehmanni* | small intestine | 11 | 0 | / | / | Qitai County  (n=11) | 2024 | Approval No. A2022-029-01 |
| *Paralaudakia caucasia* | small intestine | 16 | 0 | / | / | Qitai County  (n=16) | 2024 | Approval No. A2022-029-01 |
| *Paralaudakia microlepis* | small intestine | 10 | 0 | / | / | Qitai County  (n=10) | 2024 | Approval No. A2022-029-01 |
| Aves | | | | | | | | |
| Anseriformes | | | | | | | | |
| *Anser*  *anser* | faeces | 48 | 6  (12.5) | *Cryptosporidium* *proventriculi*  (n=6) | PV794559 | Shihezi City  (n=48) | 2024 | Approval No. A2022-029-01 |
| Lariformes | | | | | | | | |
| *Larus*  *ichthyaetus* | faeces | 20 | 1 | *Cryptosporidium* goose genotype I  (n=1) | PV794560 | Wusu City  (n=20) | 2024 | Approval No. A2022-029-01 |
| *Larus*  *fuscus* | faeces | 16 | 1 | *Cryptosporidium* goose genotype I  (n=1) | PX248577 | Wusu City  (n=16) | 2024 | Approval No. A2022-029-01 |
| *Larus*  *argentatus* | faeces | 17 | 0 | / | / | Wusu City  (n=17) | 2024 | Approval No. A2022-029-01 |
| *Larus*  *armenicus* | faeces | 5 | 0 | / | / | Wusu City  (n=5) | 2024 | Approval No. A2022-029-01 |
| *Larus*  *cachinnans* | faeces | 14 | 0 | / | / | Wusu City  (n=14) | 2024 | Approval No. A2022-029-01 |
| *Larus*  *hemprichii* | faeces | 16 | 0 | / | / | Wusu City  (n=16) | 2024 | Approval No. A2022-029-01 |
| *Charadrius*  *dubius* | faeces | 6 | 0 | / | / | Wusu City  (n=6) | 2024 | Approval No. A2022-029-01 |
| *Ciconia*  *nigra* | faeces | 2 | 0 | / | / | Wusu City  (n=2) | 2024 | Approval No. A2022-029-01 |
| *Larus*  *argentatus* | faeces | 2 | 0 | / | / | Wusu City  (n=2) | 2024 | Approval No. A2022-029-01 |
| Caprimulgiformes | | | | | | | | |
| *Caprimulgus*  *indicus* | small intestine | 12 | 0 | / | / | Shihzi City  (n=12) | 2024 | Approval No. A2022-029-01 |
| Accipitriformes | | | | | | | | |
| *Accipiter*  *nisus* | small intestine | 8 | 0 | / | / | Shihzi City  (n=8) | 2024 | Approval No. A2022-029-01 |
| *Aquila*  *chrysaetos* | small intestine | 2 | 0 | / | / | Shihzi City  (n=2) | 2024 | Approval No. A2022-029-01 |
| Passeriformes | | | | | | | | |
| *Passer*  *montanus* | small intestine | 10 | 0 | / | / | Shihzi City  (n=10) | 2024 | Approval No. A2022-029-01 |
| *Alauda*  *arvensis* | small intestine | 4 | 0 | / | / | Shihzi City  (n=4) | 2024 | Approval No. A2022-029-01 |
| *Oenanthe*  *oenanthe* | small intestine | 4 | 0 | / | / | Shihzi City  (n=4) | 2024 | Approval No. A2022-029-01 |
| *Turdus merula* | small intestine | 12 | 0 | / | / | Shihzi City  (n=12) | 2024 | Approval No. A2022-029-01 |
| *Pseudopodoces humilis* | small intestine | 3 | 0 | / | / | Shihzi City  (n=3) | 2024 | Approval No. A2022-029-01 |
| *Sturnus vulgaris* | small intestine | 2 | 0 | / | / | Shihzi City  (n=2) | 2024 | Approval No. A2022-029-01 |
| *Melanocy phamongollca* | small intestine | 4 | 0 | / | / | Shihzi City  (n=4) | 2024 | Approval No. A2022-029-01 |
| Cuculiformes | | | | | | | | |
| *Cuculus canorus* | small intestine | 5 | 0 | / | / | Shihzi City  (n=5) | 2024 | Approval No. A2022-029-01 |
| Columbiformes | | | | | | | | |
| *Streptopelia decaocto* | small intestine | 7 | 0 | / | / | Shihzi City  (n=7) | 2024 | Approval No. A2022-029-01 |
| *Streptopelia orientalis* | small intestine | 1 | 0 | / | / | Shihzi City  (n=1) | 2024 | Approval No. A2022-029-01 |
| Bucerotiformes | | | | | | | | |
| *Upupa epops* | small intestine | 3 | 0 | / | / | Shihzi City  (n=3) | 2024 | Approval No. A2022-029-01 |
| Strigiformes | | | | | | | | |
| *Strigiformes* | small intestine | 3 | 0 | / | / | Shihzi City  (n=3) | 2024 | Approval No. A2022-029-01 |
| Coraciiformes | | | | | | | | |
| *Coracias garrulus* | small intestine | 1 | 0 | / | / | Shihzi City  (n=1) | 2024 | Approval No. A2022-029-01 |
| Caprimulgiformes | | | | | | | | |
| *Caprimulgus europaeus* | small intestine | 2 | 0 | / | / | Shihzi City  (n=2) | 2024 | Approval No. A2022-029-01 |
| Struthioniformes | | | | | | | | |
| *Struthio camelus* | small intestine | 3 | 0 | / | / | Shihzi City  (n=3) | 2024 | Approval No. A2022-029-01 |
| Galliformes | | | | | | | | |
| *Pavo muticus* | small intestine | 1 | 0 | / | / | Shihzi City  (n=1) | 2024 | Approval No. A2022-029-01 |
| Gruiformes | | | | | | | | |
| *Fulica atra* | small intestine | 1 | 0 | / | / | Wusu City  (n=1) | 2024 | Approval No. A2022-029-01 |
| Total |  | 1855 |  | 150  (8.08) |  |  |  |  |
