# Supplementary material for: Investigation of Cryptosporidium infection in a broad range of hosts in northern China
Source: Parasit Vectors. 2025 Nov 26;18:509. doi: 10.1186/s13071-025-07152-9 (PMC12750556; doi:10.1186/s13071-025-07152-9)
Supplement: Supplementary file 2 — Additional file 2. Table S2. The data of (A) primers and (B) cycling conditions used in this study. The genetic markers that served for identification of species were as follows: CytB for rodent species; 16SrRNA for bird species; D-loop mtDNA for bird feces; COX1 for reptile species; SSU rRNA and GP60 for Cryptosporidium spp. [file 13071_2025_7152_MOESM2_ESM.docx]

**Additional file2: Table S2.** The data of (A) primers and (B) cycling conditions used in this study. The genetic markers that served for identification of species were as follows: *CytB* for rodent species; *16SrRNA* for bird species; D-loop mtDNA for bird feces; *COX1* for reptile species; *SSU rRNA* and *GP60* for *Cryptosporidium* spp.

(A)

| **Target taxon** | **Target**  **gene** | **Primer sequence (5’-3’)** | **Fragment size (bp)** | **Reference** |
| --- | --- | --- | --- | --- |
| Rodents | *Cytb* | L7：  ACCAATGACATGAAAAATCATCGTT  H15915：  TCTCCATTTCTGGTTTACAAGAC | 1178 bp | [20] |
| Birds | *16SrRNA* | F: CTGTAGGCCTTTAAGCAGC  R: AGGATGTCCTGATCCAACAT | 910 bp | [17] |
| Bird feces | D-loop  mtDNA | F: TCACGTGAAATCAGCAACCC  R: CATCTTCAGTGCCATGCTTT | 924 bp | [18] |
| Lizard | *COX*1 | F: TYTCWACWAAYCAYAAAGAYATCGG  R: ACYTCRGGRTGRCCRAARAATCA | 650 bp | [19] |
| *Cryptosporidium* | *SSU rRNA* | SSUrRNA-F1:  TTCTAGAGCTAATACATGCG  SSUrRNA-R1: CCCATTTCCTTCGAAACAGGA  SSUrRNA-F2: GGAAGGGTTGTATTTATTAGATAAAG  SSUrRNA-R2: AAGGAGTAAGGAACAACCTCCA | 1325 bp  826-864 bp | [21] |
|  | *GP60* | UGP-F1:  TTTACCCACACATCTGTAGCGTCG  UGP-R1:  ACGGACGGAATGATGTATCTGA  UGP-F2:  ATAGGTGATAATTAGTCAGTCTTTAAT  UGP-R2:  TCCAAAAGCGGCTGAGTCAGCATC | 1044 bp  948 bp | [22] |

(B)

| **Gene** | **Initial denaturation (℃, sec)** | **Denaturation (℃, sec)** | **Annealing (℃, sec)** | **Extension (℃, sec)** | **Cycle number** | **Final extension (℃, sec)** |
| --- | --- | --- | --- | --- | --- | --- |
| *CytB* | 94, 300 | 94, 30 | 55, 60 | 72, 60 | 30 | 72, 600 |
| *16SrRNA* | 95, 300 | 95, 30 | 55, 40 | 72, 30 | 35 | 72, 600 |
| D-loop  mtDNA | 94, 180 | 94, 30 | 59, 30 | 72, 30 | 35 | 72, 600 |
| *COX*1 | 94, 180 | 94, 30 | 50, 30 | 72, 30 | 40 | 72, 600 |
| *SSU rRNA out*  *SSU rRNA in* | 94, 300  94, 300 | 94, 45  94, 45 | 55, 45  58, 45 | 72, 60  72, 60 | 35  35 | 72, 600  72, 600 |
| *GP60 out*  *GP60 in* | 94, 300  94, 300 | 94, 45  94, 45 | 58, 45  55, 45 | 72, 60  72, 60 | 35  35 | 72, 600  72, 600 |
