# Supplementary material for: Investigation of Cryptosporidium infection in a broad range of hosts in northern China
Source: Parasit Vectors. 2025 Nov 26;18:509. doi: 10.1186/s13071-025-07152-9 (PMC12750556; doi:10.1186/s13071-025-07152-9)

**Additional file3: Fig S1.** Phylogenetic tree of *Cryptosporidium ubiquitum* and *Cryptosporidium hominis* was conducted using the maximum-likelihood method based on *GP60*gene sequences under the K2+I model with 1,000 bootstrap replicates. Novel sequences identified in this study are denoted by black circles and labeled with GenBank accession numbers, host species and country of origin.


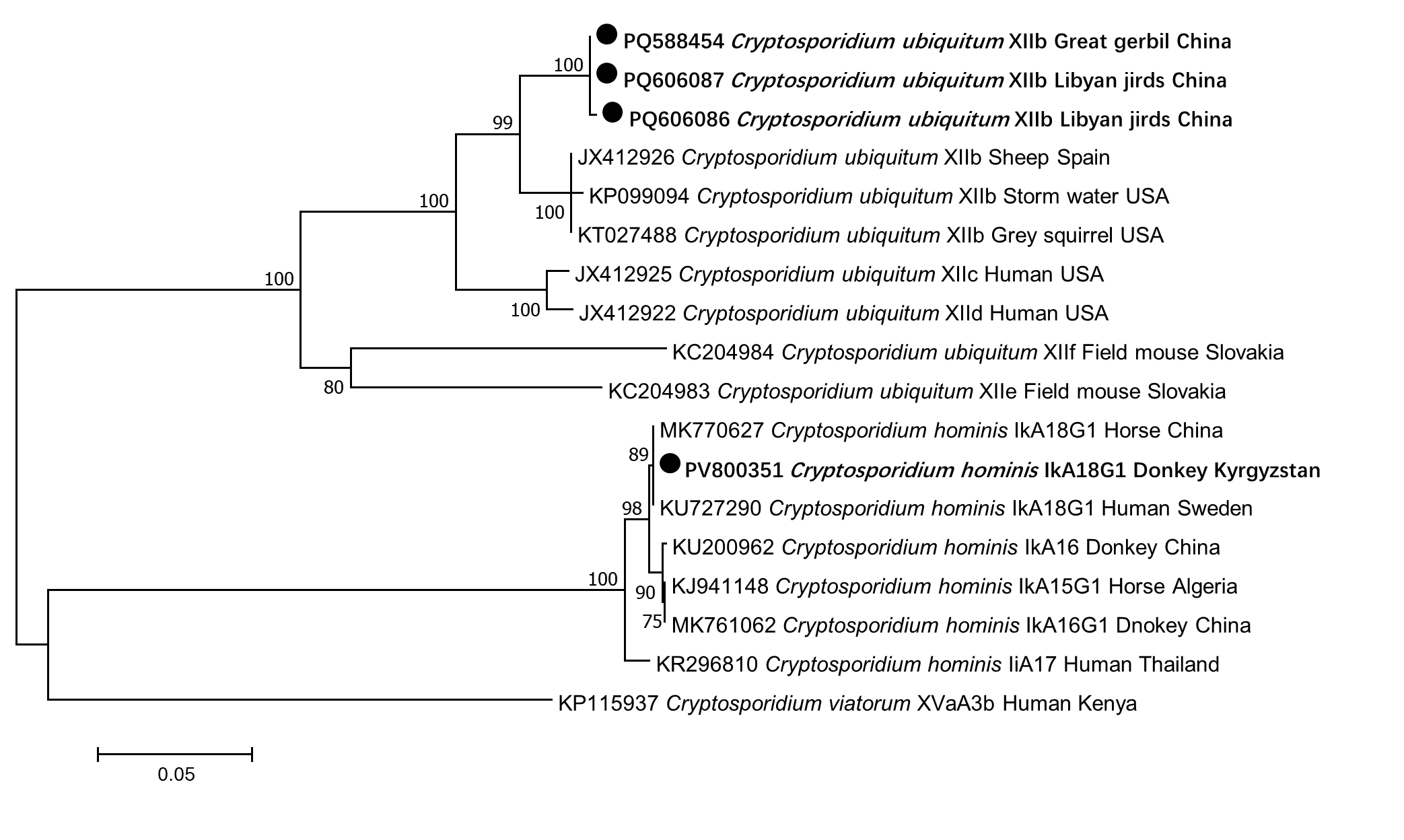

Supplement: Supplementary file 3 — Additional file 3. Figure S1. Phylogenetic tree of Cryptosporidium ubiquitum and Cryptosporidium hominis was conducted using the maximum-likelihood method based on GP60 gene sequences under the K2+I model with 1,000 bootstrap replicates. Novel sequences identified in this study are denoted by black circles and labeled with GenBank accession numbers, host species and country of origin. [file 13071_2025_7152_MOESM3_ESM.doc]
