# Supplementary material for: Investigation of Cryptosporidium infection in a broad range of hosts in northern China
Source: Parasit Vectors. 2025 Nov 26;18:509. doi: 10.1186/s13071-025-07152-9 (PMC12750556; doi:10.1186/s13071-025-07152-9)

**Additional file4: Fig S2.** Phylogenetic tree of *Cryptosporidium* was conducted using the maximum-likelihood method based on *SSUrRNA* gene sequences under the T92+I model with 1,000 bootstrap replicates.


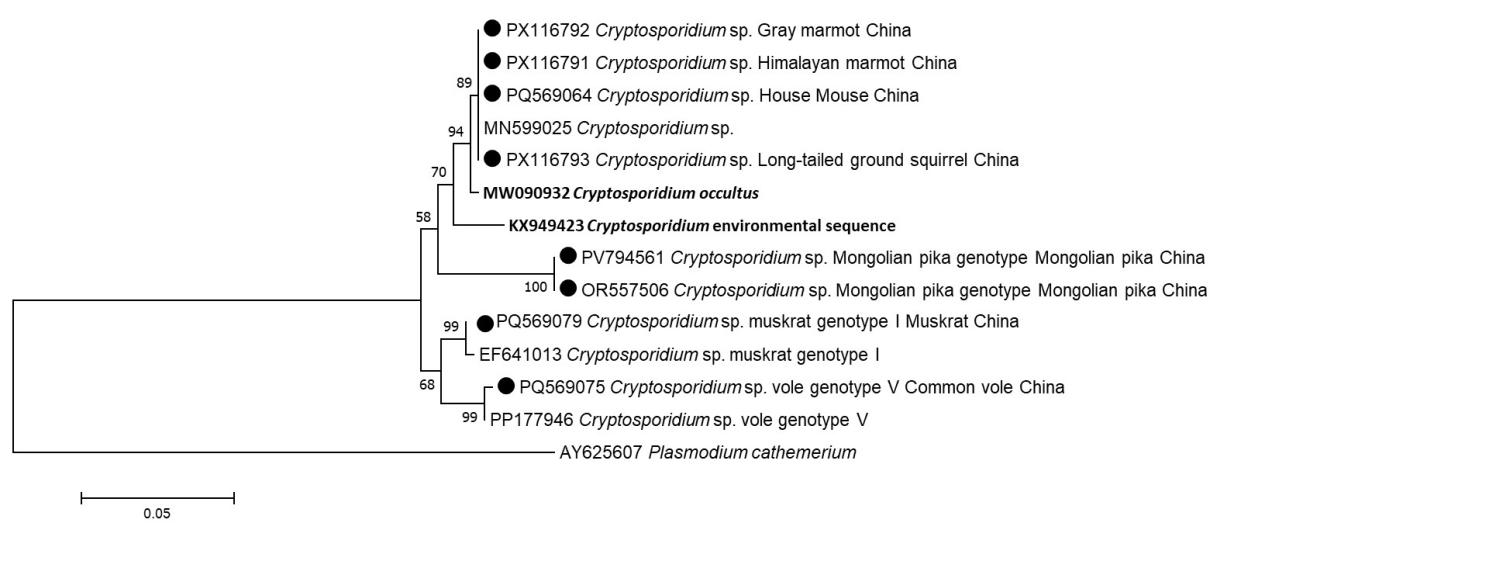

Supplement: Supplementary file 4 — Additional file 4. Figure S2. Phylogenetic tree of Cryptosporidium was conducted using the maximum-likelihood method based on SSUrRNA gene sequences under the T92+I model with 1,000 bootstrap replicates. [file 13071_2025_7152_MOESM4_ESM.docx]
